# Can the Care-need Level Determined by Local Certification Board Predict Long-term Clinical Outcomes in Patients with Stroke?

**DOI:** 10.31662/jmaj.2023-0182

**Published:** 2023-12-27

**Authors:** Kenichi Sakakura

**Affiliations:** 1 Division of Cardiovascular Medicine, Saitama Medical Center, Jichi Medical University, Saitama, Japan

**Keywords:** stroke, care-need level, outcomes

Although new treatment options including neuroendovascular technologies have been developed for stroke care ^(1)^, stroke is still associated with high morbidity and mortality ^(2)^. Most patients with stroke, specifically elderly, require public support including outpatient rehabilitation services. Moreover, in Japan, patients with stroke can receive long-term rehabilitation using long-term care insurance ^(3)^. However, a care-need level needs to be determined by the Nursing Care Needs Certification Board in services received using the long-term care insurance. A care-need level is stratified to seven levels (support level 1, support level 2, care-need level 1, care-need level 2, care-need level 3, care-need level 4, and care-need level 5). The support level 1 is the mildest, while the care-need level 5 is the most severe. Although the care-need level is widely used in Japan, there have been limited studies that validate the care-need level in the field of stroke.

Konishi et al. conducted a unique study to validate the care-need level in patients with stroke ^(4)^. They included 7491 patients from Tochigi Prefecture who underwent acute phase in-hospital rehabilitation for stroke and investigated the association between the care-need levels 6 months after discharge and long-term outcomes. In comparison to patients with support level 1, patients with higher care-need levels showed significantly higher proportions of being bedridden and higher mortality. Their study aimed to validate the care-need level by using an administrative database obtained from municipalities in Tochigi Prefecture. Most physicians in Japan are familiar with the care-need level, because the primary care physicians must fill out a paper-based statement on the patient’s condition in a standard format. However, clinical significance of the care-need level has not been fully discussed in stroke care.

One of the limitations in their study is the lack of information of the severity of stroke upon admission. However, their study suggests that the care-need level after 6 months may be clinically relevant to long-term outcomes irrespective of severity of stroke upon admission. Thus, they opened the door for the validation study regarding the care-need level in Japan. The care-need level is the key issue in the long-term care insurance system, which is supported by public funds. Therefore, future studies are warranted to validate the care-need level for stroke care in the view of cost-effectiveness.

## Article Information

### Conflicts of Interest

None
